# Supplementary material for: Kinetic sequencing (k-Seq) as a massively parallel assay for ribozyme kinetics: utility and critical parameters
Source: Nucleic Acids Res. 2021 Mar 27;49(12):e67. doi: 10.1093/nar/gkab199 (PMC8559535; doi:10.1093/nar/gkab199)
Supplement: gkab199_Supplemental_File [file gkab199_supplemental_file.pdf]

Supplementary Data

Kinetic sequencing (*k*-Seq) as a massively parallel assay for ribozyme kinetics: utility and critical parameters

Yuning Shen[1], Abe Pressman[2], Evan Janzen[1,3] and Irene A. Chen[1,3,4,*]
[1] Department of Chemistry and Biochemistry, University of California, Santa Barbara, California 93106, United States
[2] Department of Chemical Engineering, University of California, Santa Barbara, California 93106, United States
[3] Program in Biomolecular Sciences and Engineering, University of California, Santa Barbara, California 93106, United States
[4] Department of Chemical and Biomolecular Engineering, Department of Chemistry and Biochemistry, University of California, Los Angeles, California 90095, United States

Present Address: Abe Pressman, Cellular Engineering Group, National Institute of Standards and Technology, 100 Bureau Drive, Gaithersburg, Maryland 20899, United States

Table S1. Wild-type sequences selected from (1) for the variant pool

#	Ribozyme	Sequence (random region)
1	S-2.1-a	ATTACCCTGGTCATCGAGTGA
2	S-1A.1-a	CTACTTCAAACAATCGGTCTG
3	S-1B.1-a	CCACACTTCAAGCAATCGGTC
4	S-3.1-a	AAGTTTGCTAATAGTCGCAAG

Figure S1. Standard curve for qPCR measurement, fitted by ‘stats.linregress’ function from ‘SciPy’ Python package.

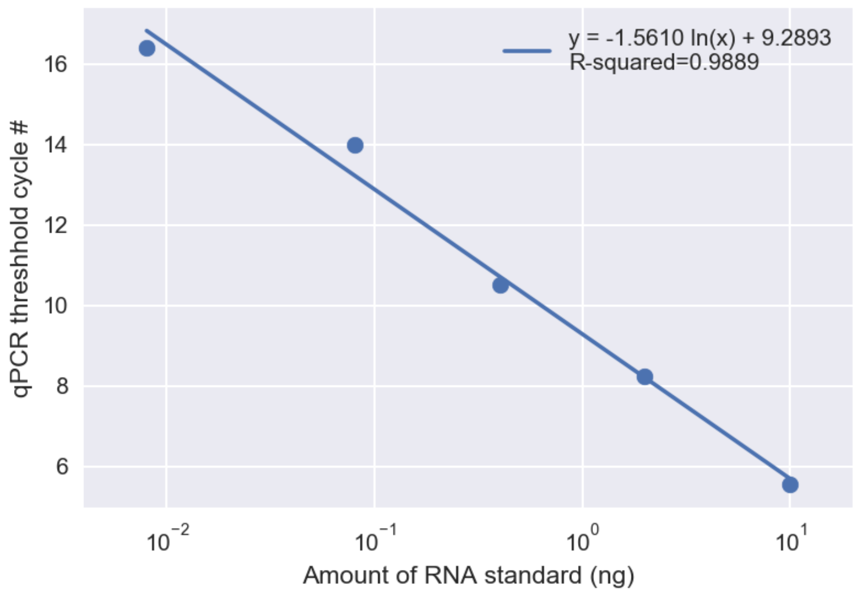

Figure S2. Relative standard deviation vs. mean of total RNA measured for each set of reacted sample triplicates, quantified by spike-in sequence (circles) or qPCR + Qubit (crosses). The mean relative standard deviations are similar for spike-in method (0.165) and for qPCR + Qubit method (0.176).

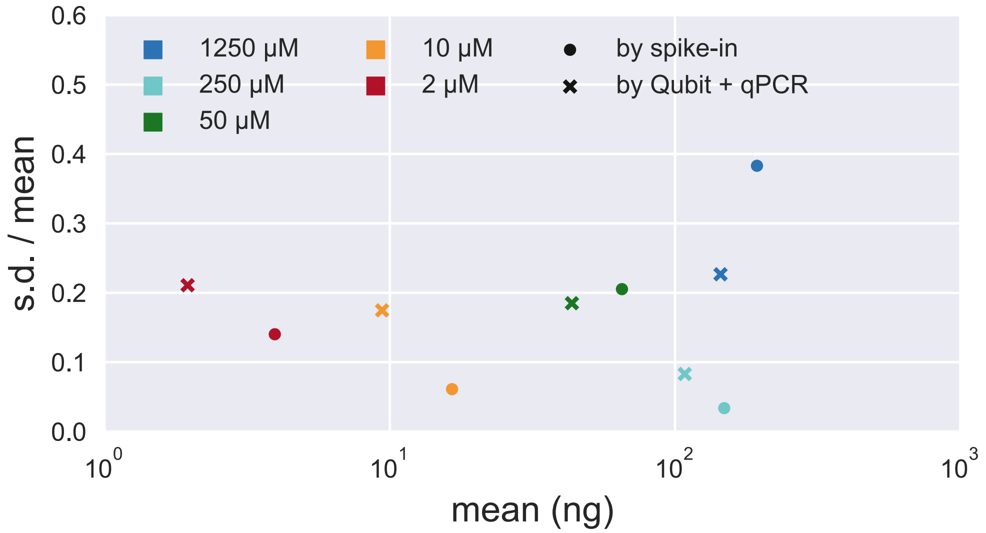

Text S1. Using customized kinetic models in the ‘k-seq’ package. Kinetic models with a closed form integrated rate law can be written as a python function, with the independent variables and parameters to be estimated as the input arguments, and the dependent variables as the return values. The function can be passed to the `k_seq.estimate` module for *k*-Seq fitting.

In general, rate constants can be determined by varying either time points or concentrations. The choice depends on experimental concerns. In the case of the aminoacylation ribozymes studied in this work, varying the initial concentration of substrate BYO was experimentally desirable for the following reasons. First, because BYO hydrolyzes in aqueous solution, fixing the time point simplified the form of the kinetic model for fitting (namely, introducing a constant factor to the exponential rather than a time-dependent exponential of an exponential; please see Supporting Text S3 in (1) for details). Second, in practice, the spin column purification step took about 5 min, which prevented accurate sampling at early times. Instead, a longer time (90 min) could be used at low substrate concentrations to probe the same activity regime. While substrate degradation or a need for timing accuracy may favor use of a concentration series, a time series may be desirable in other circumstances, such as if the amount of ribozyme or substrate is limited. The ‘k-seq’ package may be used to fit either type of data by passing the appropriate function.

As another example, *k*-Seq data can be fit to second-order kinetics. The integral form for second-order kinetics can be expressed as

$$\ln\frac{s_0 - s_t}{s_0} - \ln\frac{c_0 - s_t}{c_0} = -(c_0 - s_0)kt$$

where s_0 is the initial amount of the sequence, s_t is the amount of reacted sequence at time t, c_0 is the initial substrate concentration, and k is the second-order rate constant. Let $s_t = fs_0$ where f is the reacted fraction. The reacted fraction f (as the dependent variable measured in *k*-Seq experiment) can be written as:

$$f = 1 - \frac{(c_0 - s_0)e^{-(c_0-s_0)kt}}{c_0 - s_0 e^{-(c_0-s_0)kt}}.$$

This function can be used to fit the *k*-Seq experiments with varying time t or varying initial substrate concentration c_0, and a maximum amplitude may be added as a parameter if desired.

Figure S3. Illustration of the 6 regions selected to sample sequences and their fitting values from simulated reacted fraction dataset, with the boundary values for true A, k, and kA indicated in the table (N/A = not applicable). These regions are separately analyzed in Figure S4-S9.

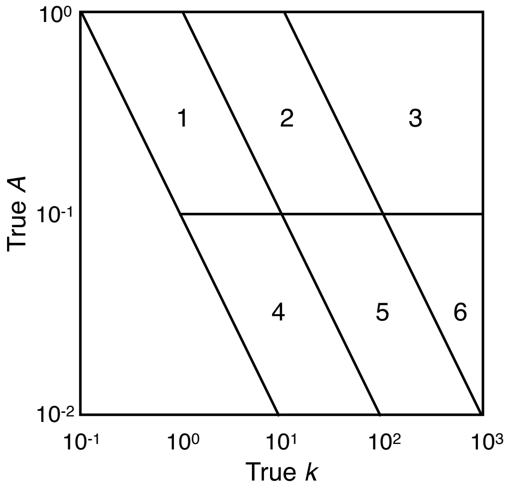

Region	k $(\mathrm{min}^{-1}\mathrm{M}^{-1})$	A	kA $(\mathrm{min}^{-1}\mathrm{M}^{-1})$
1	N/A	$10^{-1} < A < 1$	$10^{-1} < kA < 1$
2	N/A	$10^{-1} < A < 1$	$1 < kA < 10^{1}$
3	$k < 10^{3}$	$10^{-1} < A < 1$	$kA > 1$
4	N/A	$10^{-2} < A < 10^{-1}$	$10^{-1} < kA < 1$
5	N/A	$10^{-2} < A < 10^{-1}$	$1 < kA < 10^{1}$
6	$k < 10^{3}$	$10^{-2} < A < 10^{-1}$	$kA > 1$

Figure S4. Selected fitting results from Region 1 ($0.1 < A < 1$, $0.1 < kA < 1\ \mathrm{min^{-1}M^{-1}}$) in simulated reacted fraction dataset with different relative error. Each curve plot shows the reacted fraction (in triplicates) at various initial BYO concentrations (orange crosses), fitting curves from point estimation (blue line), and fitting curves from 20 repeated fitting or 20 bootstrapped samples (grey lines); fitted k, A values for the curves are shown in the corresponding heatmap (red crosses) under each curve plot. For visual guidance, background color of the heatmap indicates the relative values of mean squared error (normalized in each plot; blue to yellow is lower to higher error) over the parameter space given the data. The white dashed line marks $\mathrm{kA} = 1\ \mathrm{min^{-1}M^{-1}}$. An ideal fitting result would have converged fitting optima and is both numerically stable (from repeated fitting) and robust to noise (from bootstrapping). A large variance along the line of $kA = \mathrm{constant}$ indicates the model is not identifiable, i.e., k and A cannot be separately estimated. (react. frac. = reacted fraction.)

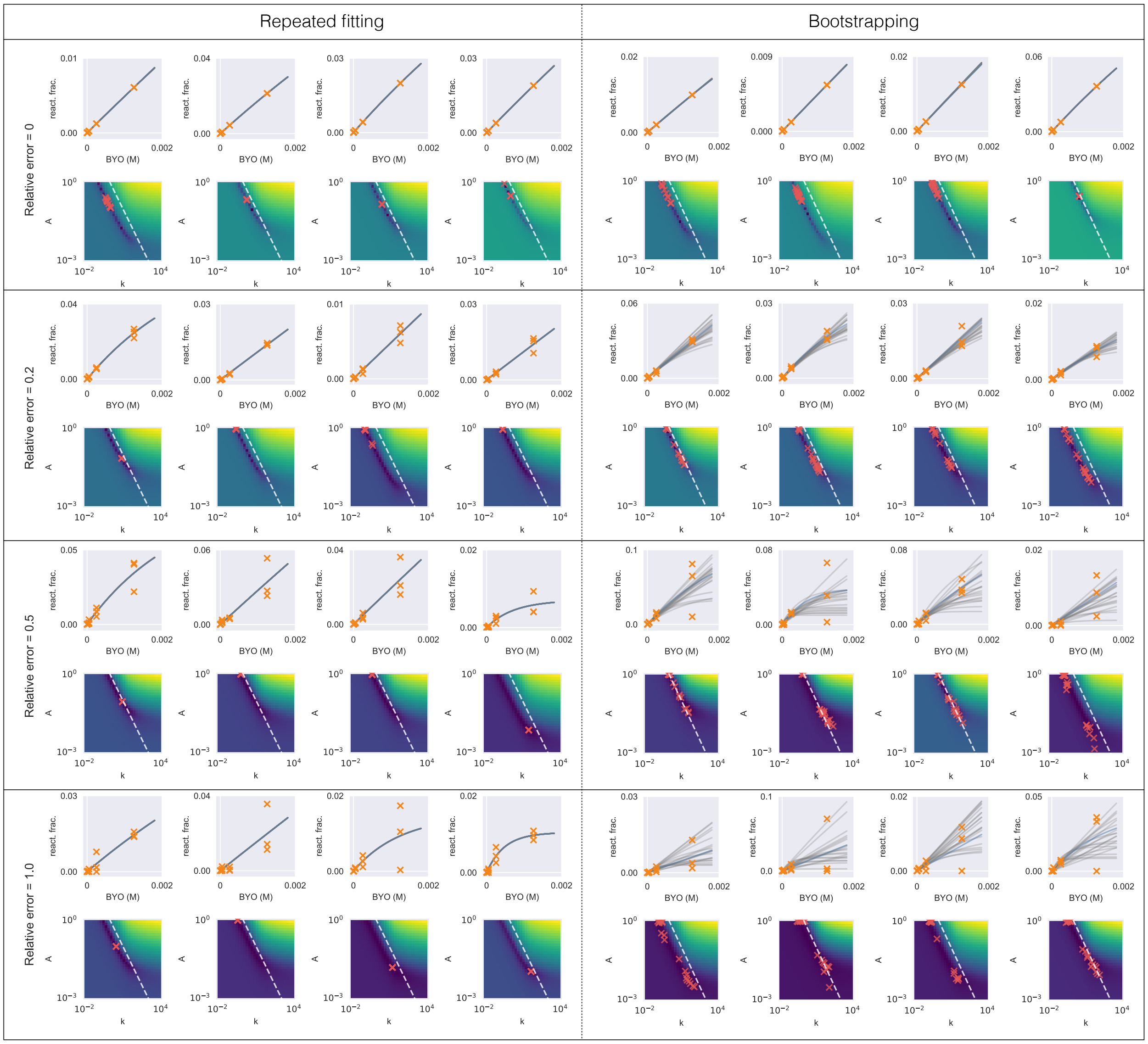

Figure S5. Selected fitting results from Region 2 ($0.1 < A < 1, 1 < kA < 10\ \mathrm{min}^{-1}\mathrm{M}^{-1}$) in simulated reacted fraction dataset with different relative error. See caption of Figure S4 for explanation.

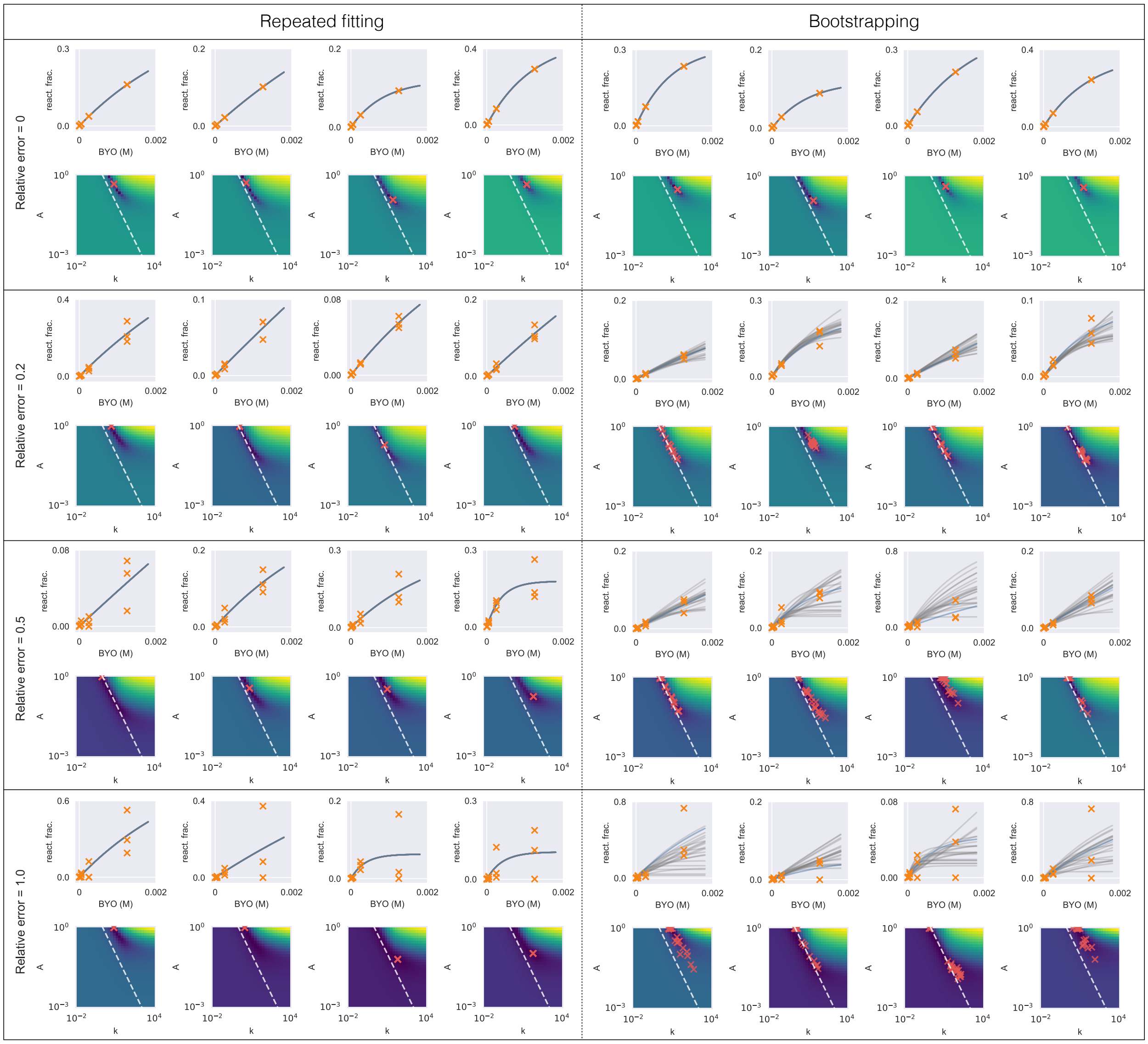

Figure S6. Selected fitting results from Region 3 ($0.1 < A < 1, kA > 10 \text{ min}^{-1}\text{M}^{-1}$) in simulated reacted fraction dataset with different relative error. See caption of Figure S4 for explanation.

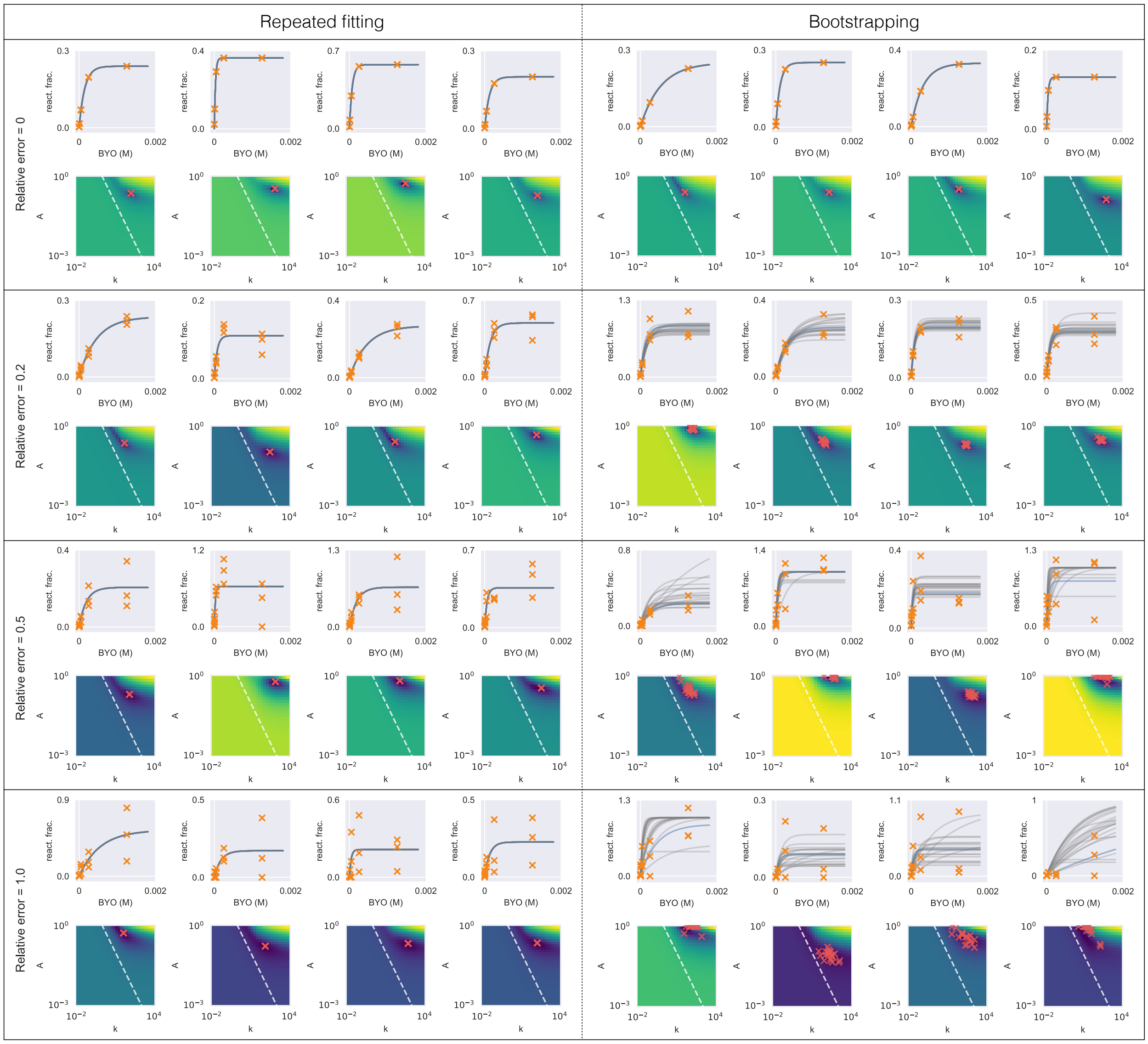

Figure S7. Selected fitting results from Region 4 ($A < 0.1$, $0.1 < kA < 1\ \mathrm{min}^{-1}\mathrm{M}^{-1}$) in simulated reacted fraction dataset with different relative error. See caption of Figure S4 for explanation.

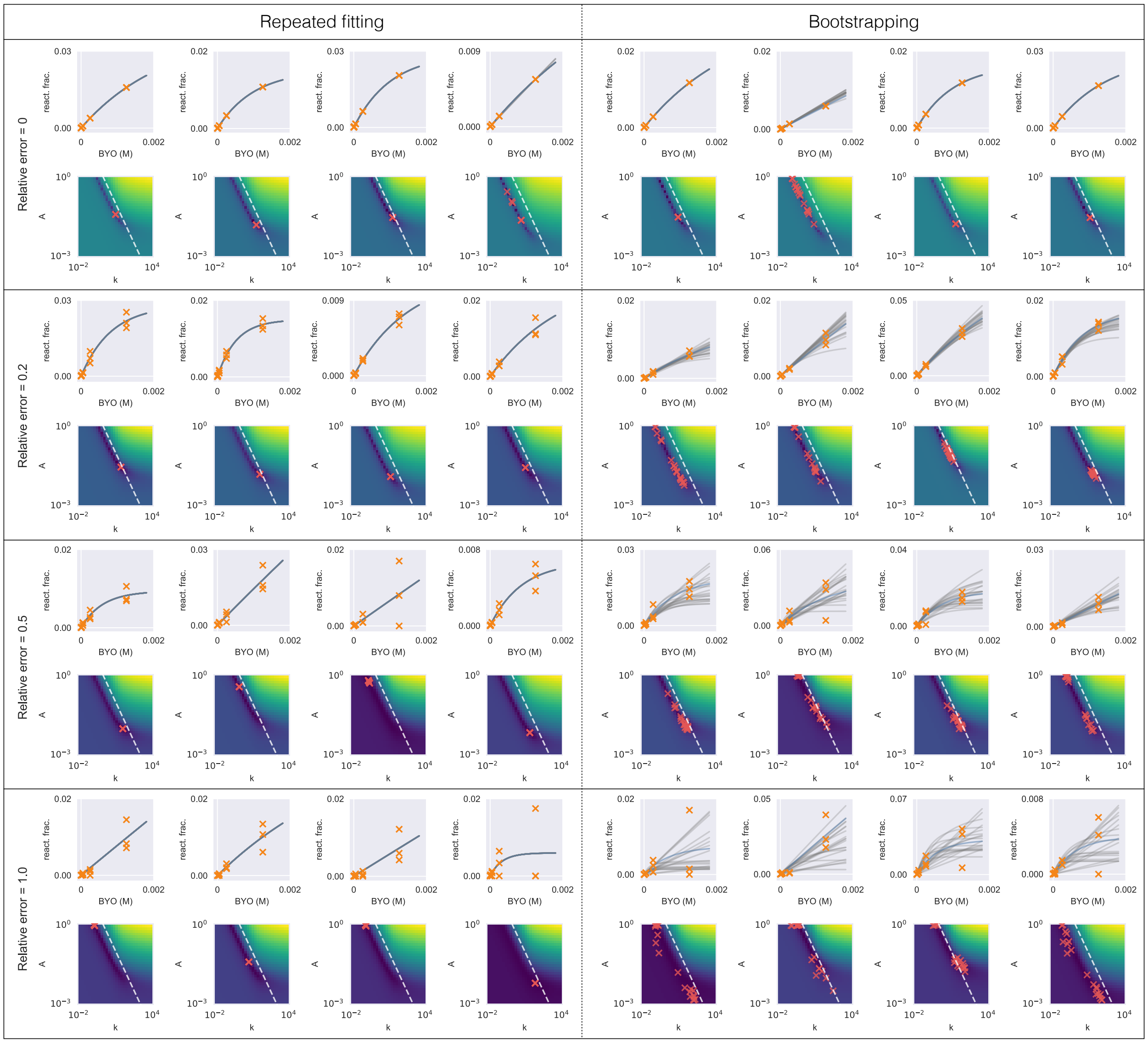

Figure S8. Selected fitting results from Region 5 ($A < 0.1$, $1 < kA < 10\ \mathrm{min}^{-1}\mathrm{M}^{-1}$) in simulated reacted fraction dataset with different relative error. See caption of Figure S4 for explanation.

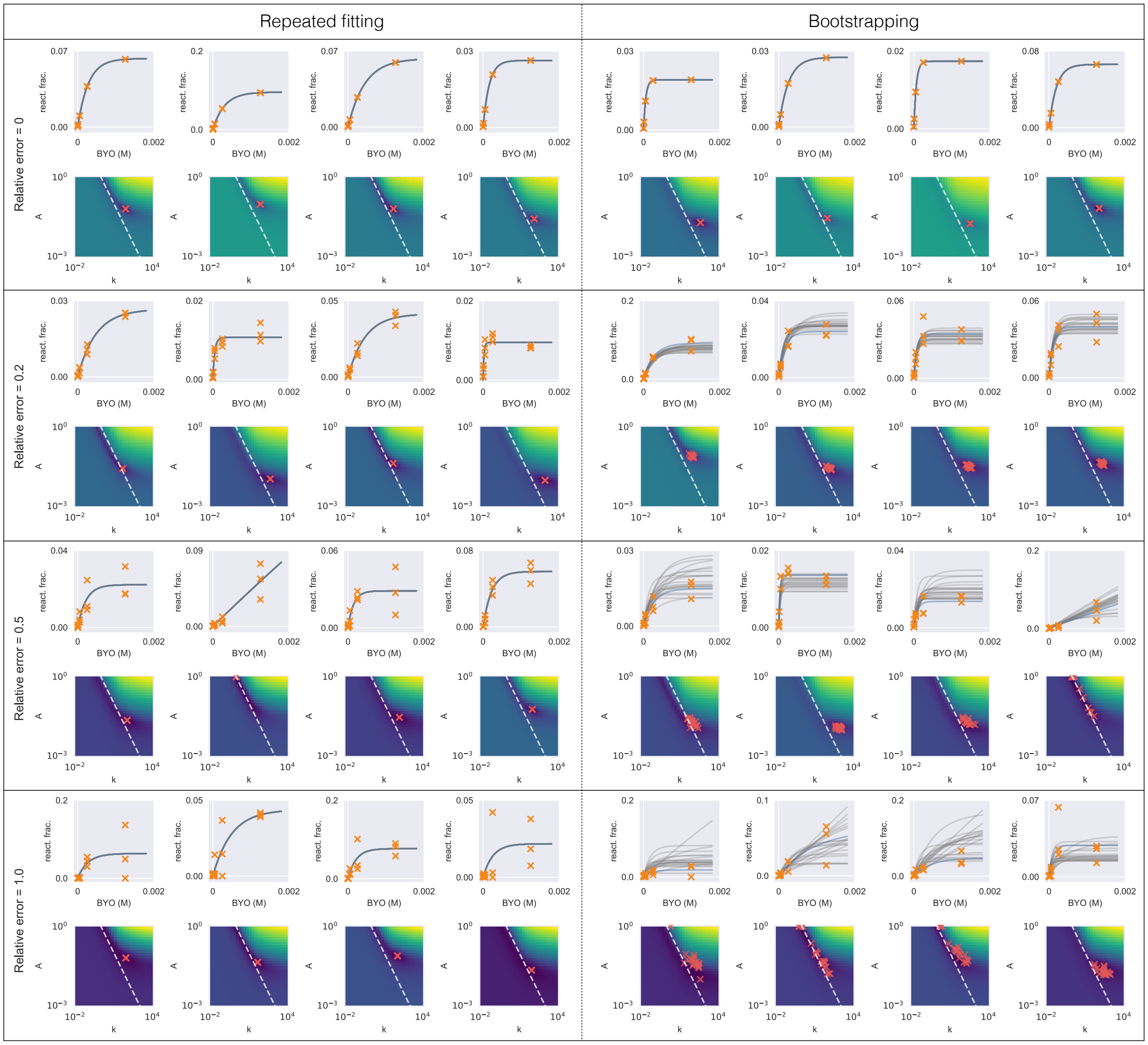

Figure S9. Selected fitting results from Region 6 ($A < 0.1$, $kA > 10\ \mathrm{min}^{-1}\mathrm{M}^{-1}$) in simulated reacted fraction dataset with different relative error. See caption of Figure S4 for explanation.

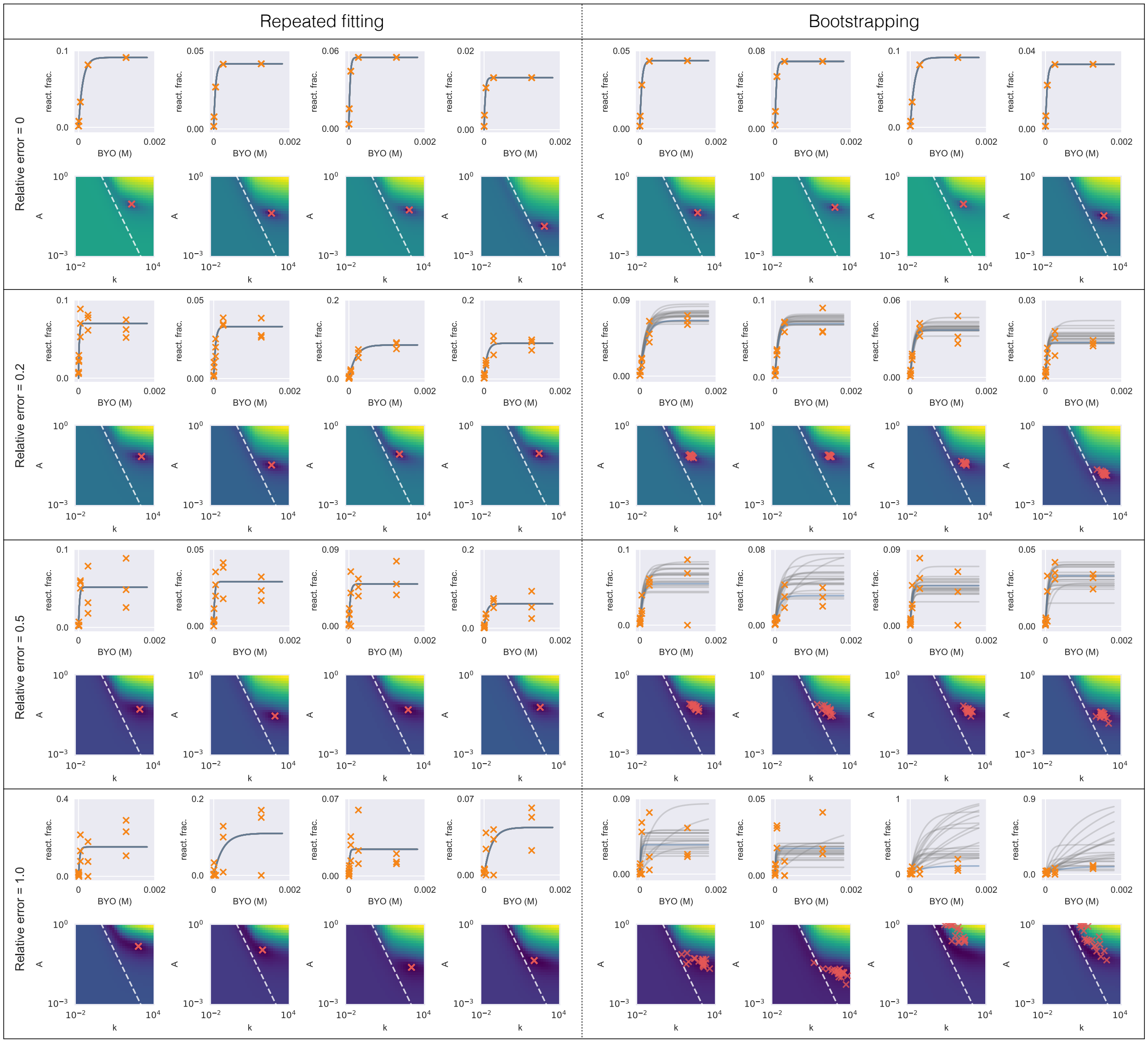

Table S2. Summary of visual examination of model identifiability from repeated fitting (no resampling) and bootstrapping. 'Y' indicates regions where k and A appear that they can be separately estimated, 'N' indicates regions where they do not appear to be separately estimable. Results from repeated fitting account for the numeric effect from different initial values and results from bootstrapping also account for the effect of sample noise.

Method	Relative error (ϵ)	Region 1	Region 2	Region 3	Region 4	Region 5	Region 6
Repeated fitting	0.0	N	Y	Y	N	Y	Y
	0.2	Y	Y	Y	Y	Y	Y
	0.5	Y	Y	Y	Y	Y	Y
	1.0	Y	Y	Y	Y	Y	Y
Bootstrapping	0.0	N	Y	Y	N	Y	Y
	0.2	N	N	Y	N	Y	Y
	0.5	N	N	N	N	N	N
	1.0	N	N	N	N	N	N

Figure S10. Distribution of metric values for sequences from 6 selected regions from the simulated reacted fraction dataset, with various noise level. Histogram bars are stacked for visibility. Both metrics σ_A and γ captured the trend of model identifiability as summarized in Table S2. In contrast, ΔA failed to capture the difference in model identifiability between sequences with different regions and sample noise.

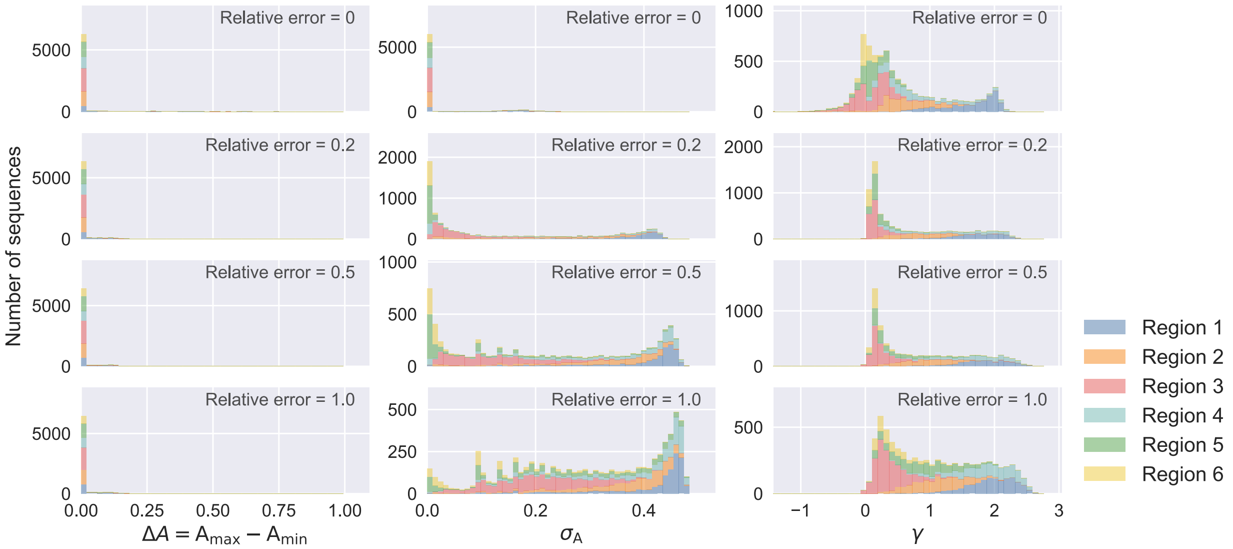

Figure S11. Distribution of γ (A) and σ_A (B) for sequences within Hamming distance of 2 to the family centers from the variant pool *k*-Seq experiment. Example fitting results are shown for sequences within each score range (labels on the left) of γ (C) and σ_A (D). For explanation of (C) and (D), also see caption of Figure S4. Sequences with low metric scores for both metrics showed good model identifiability while k and A cannot be separately estimated for those with high metric scores. k and A for most sequences up to double mutants could not be estimated separately.

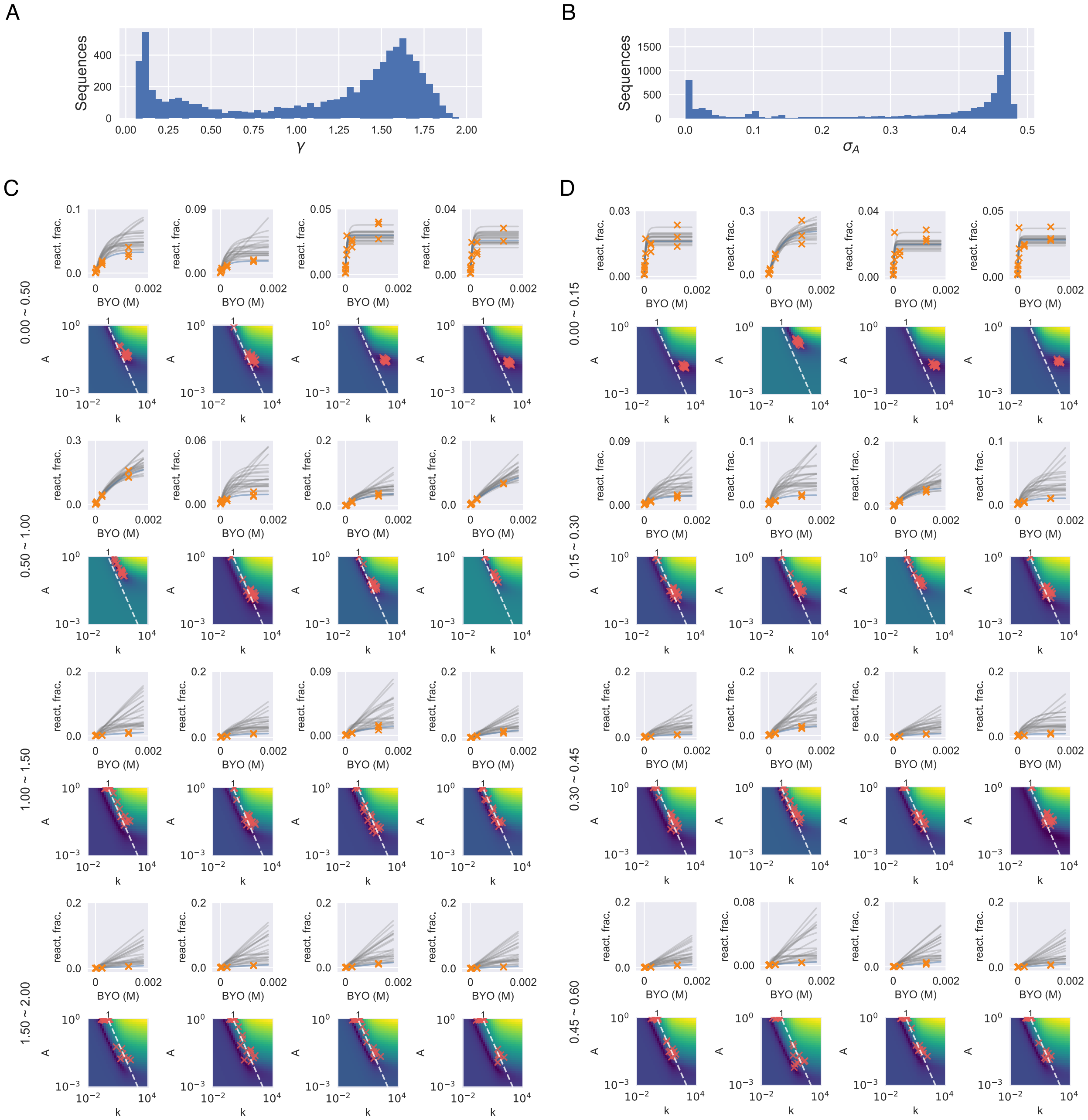

Figure S12. Correlation between model identifiability metrics γ and σ_A for analyzable sequences in the variant pool *k*-Seq experiment. Sequences within Hamming distance of 2 to the family centers ($d \leq 2$) showed good correlation between the two metrics (Spearman's ρ = 0.945, p-value = 0.000); larger variance was observed for sequences with $d > 2$ due to lower counts and noisier measurements.

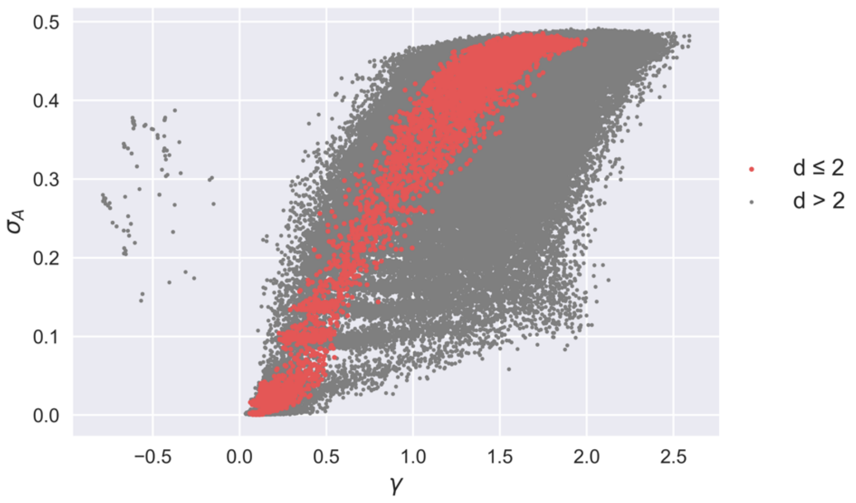

Figure S13. Processing of sequence reads. (A) Number of raw paired-end reads in each sample. The input sample (unreacted) had 3x of other samples for total DNA input for sequencing. (B) Percent of total reads retained after paired-end reads joining, filtering (removal of spike-in sequence and sequences that are not 21 nt long or with ambiguous nucleotides 'N'), and checking for analyzability (has non-zero counts in the input pool and in at least one of the reacted samples)

A

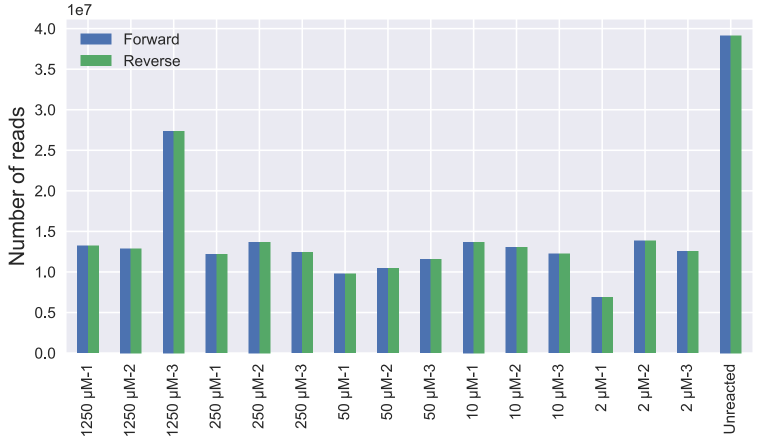

B

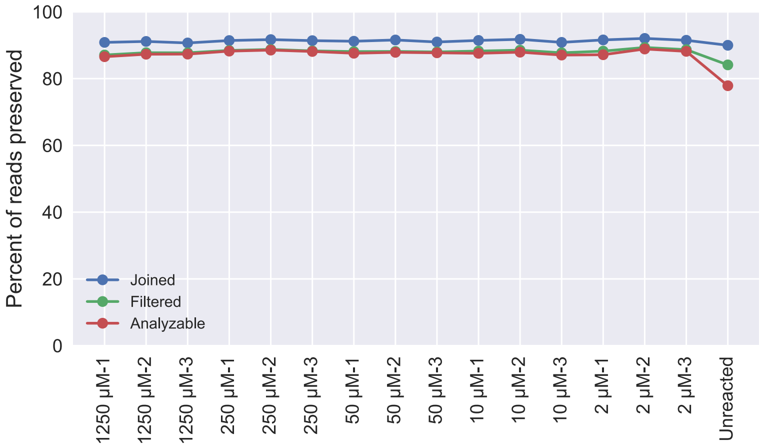

Table S3: Categorization of analyzable and non-analyzed sequence reads. We used a stringent criterion for joining, namely exact matching of the sequence reads in both directions. While this resulted in a substantial fraction of discarded reads, the benefit of improved sequencing error was important. The next largest category was sequences detected in the unreacted sample but not in the reacted samples; these are presumed to be relatively inactive sequences and tended to be higher-order mutants. To check that the analyzability requirement did not miss highly reactive sequences that were under-represented in the unreacted pool, we determined the mean counts for sequences that were found in the reacted samples but not in the unreacted sample. The highest mean count for those sequences was 3.6 reads, indicating that these sequences were not seen at high enough copy number after reaction to be considered a reliable candidate as an active sequence. However, the possible appearance of such candidates would depend on the specific experimental design and ribozyme being studied.

	Unreacted sample	Reacted samples (s.d.)
Analyzable	77.9%	87.7% (0.6%)
Failed joining of paired-end reads (perfect match required)	10.0%	8.7% (0.4%)
Spike-in sequence	0.5%	1.2 (0.3%)
Failed quality control (required 21-nt region with no ‘N’ nucleotides)	5.4%	1.9 (0.3%)
Not detected in other sample	6.2% (not detected in a reacted sample)	0.5% (0.2%) (not detected in the unreacted sample)

Text S2. Variant pool design.

We defined a sequence with *d* substitutions (Hamming distance) compared to the wild-type sequence as a *d*-th order mutant. For each partially mutated library (a single family) with randomized length *L*=21 residues and mutation rate η, the fraction of a *d*-th order mutant in the pool is

$$p_d = (1-\eta)^{L-d} \left(\frac{\eta}{3}\right)^d \qquad \text{(S1)}$$

The η maximizing the fraction of a single *d*-th order mutant satisfies $dp_d/d\eta = 0$, thus

$$\eta_d = \arg\max_\eta p_d = d/L \qquad \text{(S2)}$$

and the maximum fraction for a *d*-th order mutant in the variant pool is

$$p_{d,\max} = \left(1-\frac{d}{L}\right)^{L-d} \left(\frac{d}{3L}\right)^d \qquad \text{(S3)}$$

We used Equation S3 to determine the optimal η maximizing the fraction of a given order of mutant in the pool. For a sample with N total reads, the expected counts for a *d*-th order mutant is $p_d N$.

Figure S14. Expected counts for mutants in mixed variant pools of four wild types given different mutation rate (η) and 10^7 total reads, calculated using Equation S1. For mutants with $d > 2$, the maximum number of expected counts is not sufficient for all possible *d*-th order mutant sequences to be estimated accurately (i.e., counts are < 10-100 at this sequencing depth), showing a limitation of the variant pool library.

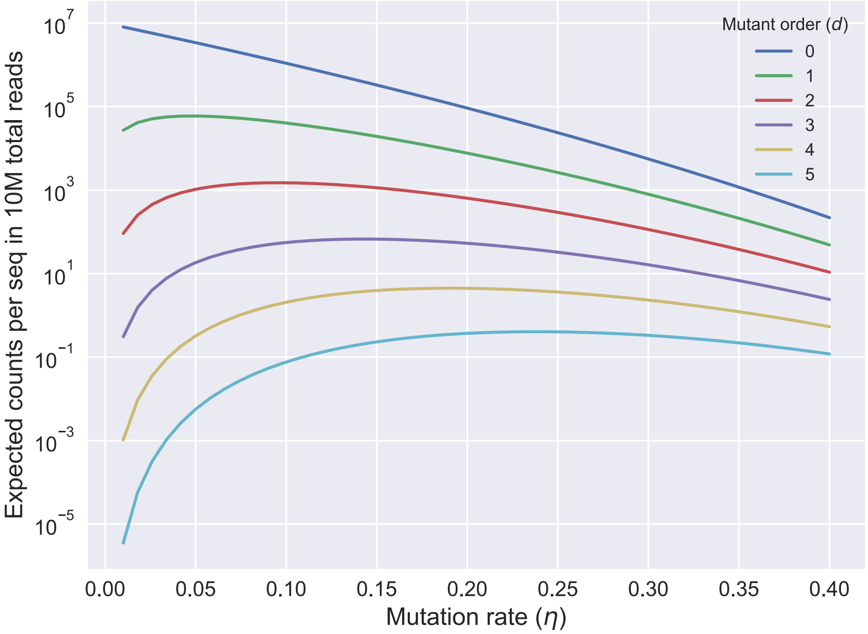

Text S3. Effects of sequencing error in the variant pool.

From Equation S1, the relative abundance ratio between a *d*-th mutant and a (*d+1*)-th mutant is

$$\phi = p_d/p_{d+1} = \frac{3(1-\eta)}{\eta} \quad \text{(S4)}$$

Assuming a constant sequencing error rate ξ per nucleotide, and considering the effect of sequencing error only by substitution, the probability that a sequence will be misidentified as one of its one-mutation neighbors is

$$(1-\xi)^{L-1}\frac{\xi}{3} \quad \text{(S5)}$$

A *d*-th order mutant, it has $3L$ neighbors consisting of d (*d*-1)-th mutants (i.e., one of the *d* mutated nucleotides reverted to the wild type), $2d$ *d*-th mutants (i.e., one of the *d* mutated nucleotides changed to one of other two possible mutations), and $3(L-d)$ (*d+1*)-th mutants (i.e., one of the $L-d$ wild type nucleotides mutated). Assuming the real abundance for this *d*-th order mutant is 1, the (*d-1*)-th mutant is ϕ, and (*d+1*)-th mutant is $1/\phi$. The expected observed abundance for a *d*-th mutant, in a variant pool with mutation rate η and sequencing error rate ξ is

$$\rho(d, \xi, \eta) = (d\phi + 2d + (3L-3d)/\phi)(1-\xi)^{L-1}\frac{\xi}{3} + (1-\xi)^L \quad \text{(S6)}$$

The fraction of abundance that originates from its neighbors due to sequencing error is

$$1 - \frac{(1-\xi)^L}{\rho(d,\xi,\eta)} \quad \text{(S7)}$$

Figure S15. Expected error from single-mutation neighbor sequences due to sequencing errors, for different orders of mutants (d) and different rates of sequencing error (ξ). Family centers $(d = 0)$ are the most abundant sequences and would be least affected by sequencing error. With decreased error rate, the fraction of reads resulting from erroneous reads of neighboring sequences is decreased for each order of mutants. The mutation rate in synthesizing the variant pool is 9%.

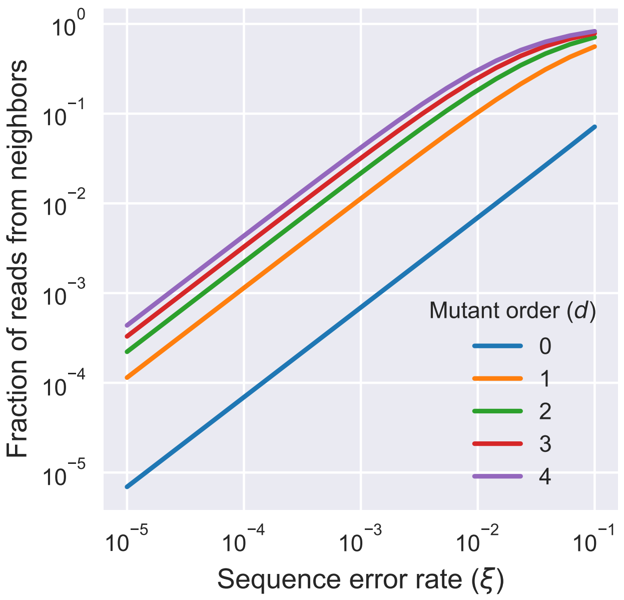

Figure S16. Alignment of gel-shift assay results for selected sequences (kA between 50 ~ 700 min^{-1}M^{-1}, measured by gel-shift) reported in (1) against the *k*-Seq results of the variant pool reported in this work. Data from the gel-shift assay were fit following the same schema as the *k*-Seq experiments: for each sequence, reacted fractions from all replicates were pooled for point estimation and bootstrapping (100 samples). The gel-shift results and the *k*-Seq results were each normalized to baseline activity to calculate the catalytic enhancement (1). Seven sequences were found to be analyzable in the variant pool, including the most and the least active sequences among the ten measured. A Pearson correlation of 0.835 (p-value = 1.94×10^{-2}) and a Spearman correlation of 0.750 (p-value = 5.22×10^{-2}) were found between the *k*-Seq measured value from the variant pool and the gel shift-measured value. The results from two *k*-Seq experiments (analyzing the variant pool reported here and the enriched pool reported in (1)) were also well-correlated with a Pearson correlation of 0.904 (p-value = 5.16×10^{-3}) and a Spearman correlation of 0.821 (p-value = 2.34×10^{-2}). Thus, estimated kA was similar among *k*-Seq experiments and the gel-shift assay. The circular markers represent the point estimates of kA and the error bars indicate standard deviation estimated from bootstrapping.

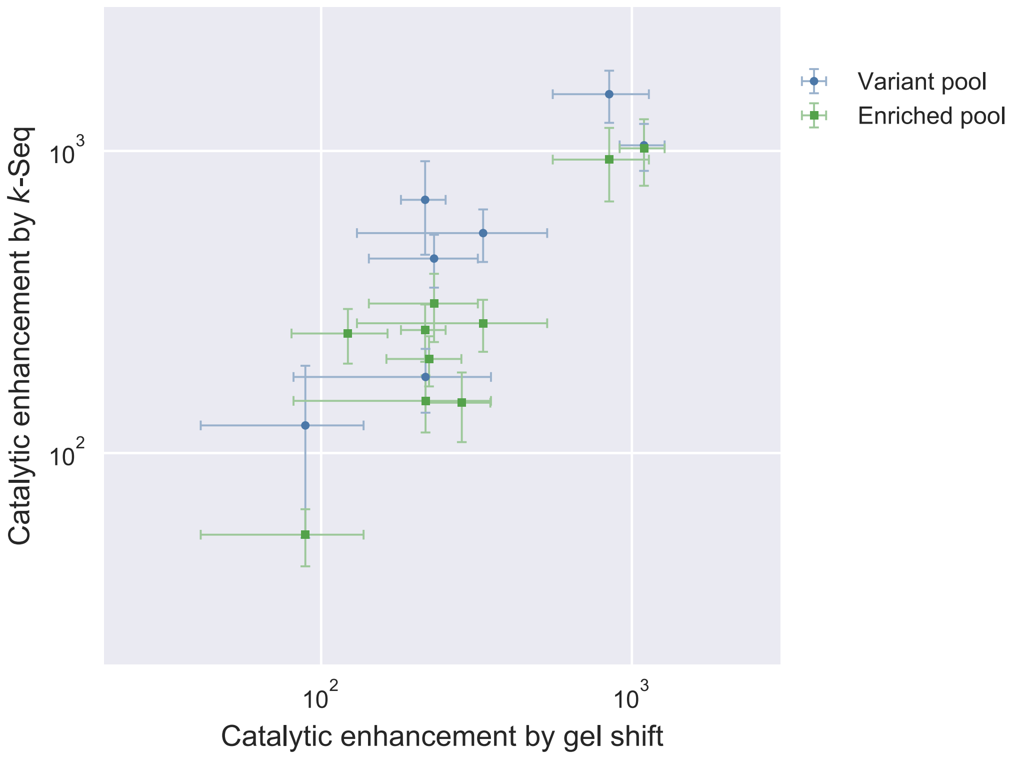

Figure S17. Fraction of sequences in which the estimated CI-95 (from bootstrapping or triplicates) includes the true kA values, for sequences with different true kA values. Sequences were ranked by true kA values (from large to small) and each data point indicates the fraction of CI-95 that include the true value in each bin of 25,000 sequences. While CI-95 estimates from bootstrapping consistently includes ~95% of true values, results from triplicates underestimate the uncertainty (i.e., over-confidence), especially for sequences with high kA values.

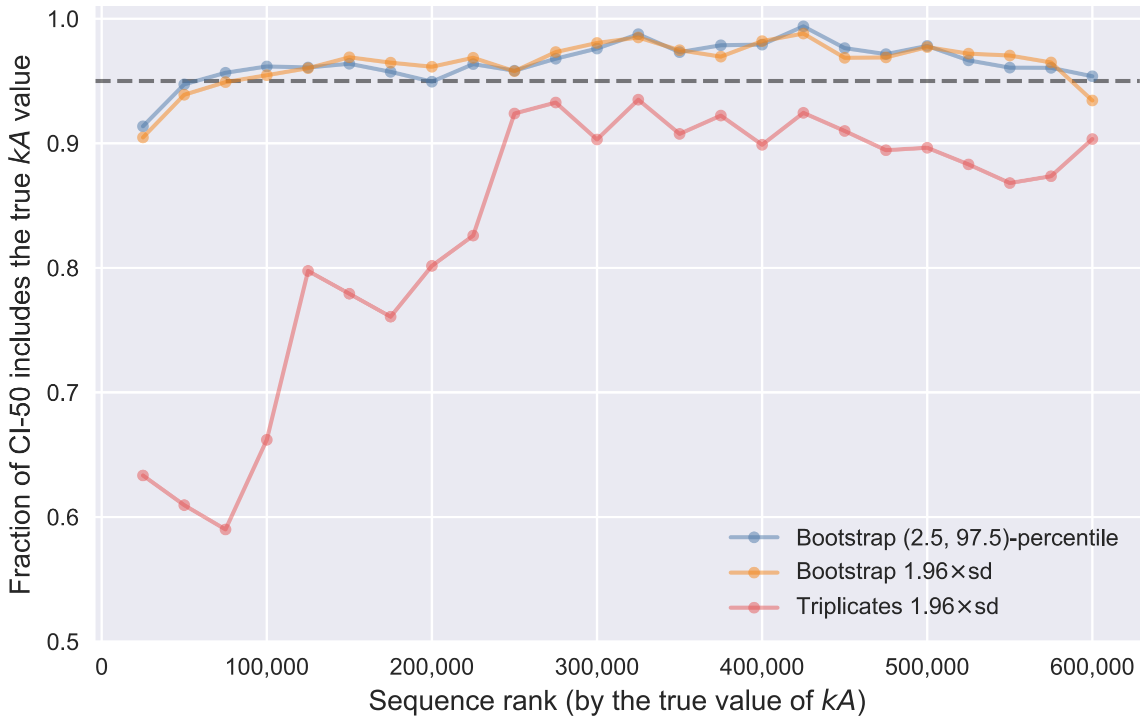

Figure S18. Precision (fold-range: 97.5-percentile / 2.5-percentile) did not have a strong dependence on kA (median of bootstrapping samples). A mild decrease to a plateaued was observed as median kA increases. Each dot represents a sequence, colored by Hamming distance d to the family center (see legend).

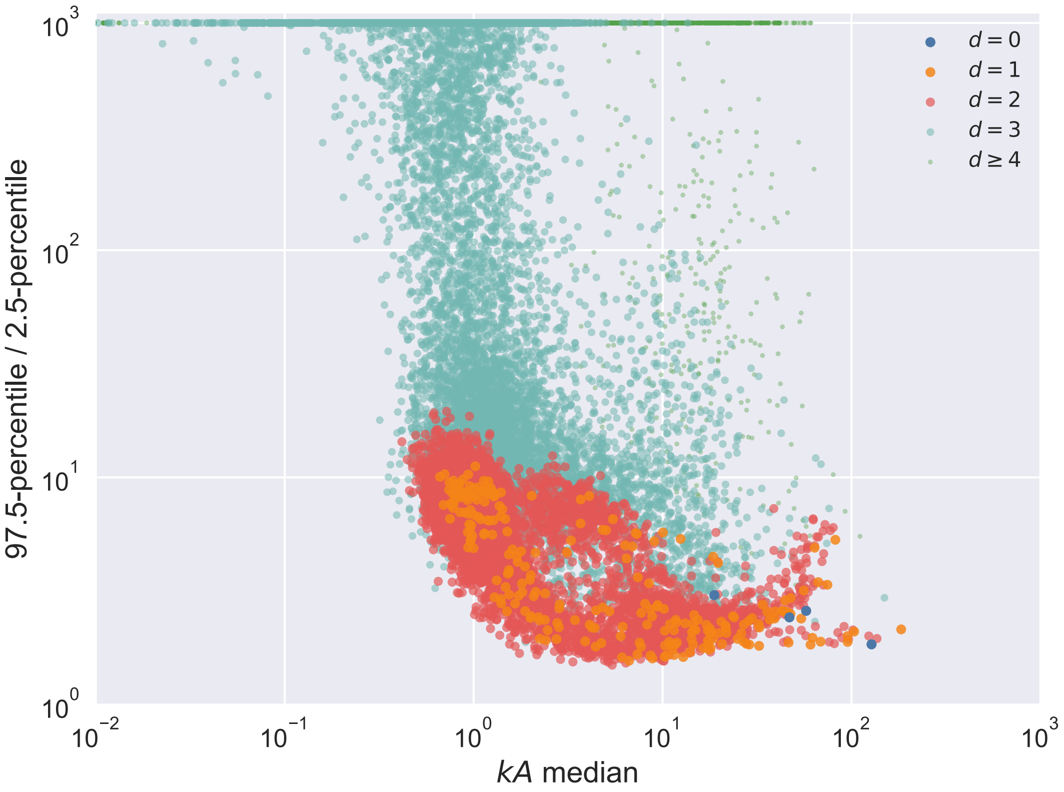

Figure S19: Low amount of non-specific RNA binding. A gel shift assay was performed by incubating 100 pmol of library RNA containing a random variable region (N = 25% A/G/C/T) with specified concentrations (in µM) of BYO or BFO (biotinyl-phenylalanyl-oxazolone) in 50 µL selection buffer for 90 minutes (1). After desalting, 5 µL streptavidin (New England Biolabs) was added to each sample, which were then incubated for 30 minutes, and analyzed by 8% native PAGE with SYBR Gold staining (Invitrogen). No quantifiable signal was seen on the gel without substrate, consistent with Figure 3B and S6A in reference (1). In addition, the fraction of RNA recovered by streptavidin beads in the absence of substrate was also quantified by HTS. A sample containing 100 pmol of RNA (a selected pool containing the four families studied here) was incubated in buffer without substrate for 90 min. RNA bound to the streptavidin beads was isolated, and 43 fmol of the spike-in sequence were added for quantification. The RNA was reverse-transcribed and the DNA was sequenced by HTS to quantify the amount of non-spike-in sequence (edit distance to the spike-in sequence > 2). We recovered a fraction of ~ 1.3×10^{-5} of the initial RNA in the absence of substrate, compared to a fraction of ~ 1.8×10^{-3} recovered at the lowest concentration of BYO (2 µM) in the variant pool *k*-Seq experiment. Thus, the amount of RNA non-specifically bound to the beads is approximately two orders of magnitude less than the smallest amount of RNA recovered in this concentration series.

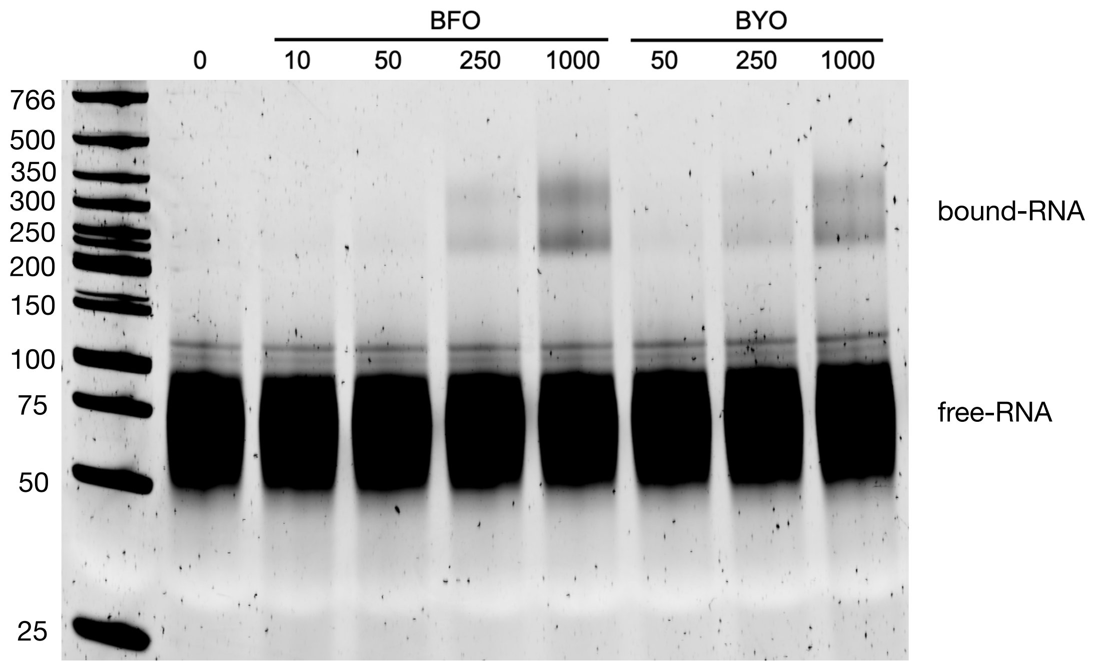

Figure S20. Dependence of accuracy (ratio of estimated kA over true kA) on counts in the unreacted pool, from simulated count data. The dashed lines correspond to ratios as labeled. Ratios above 100-fold or below 0.01-fold are shown at the borders. The counts of the simulated pool have an uneven distribution similar to the unreacted sample of the variant pool.

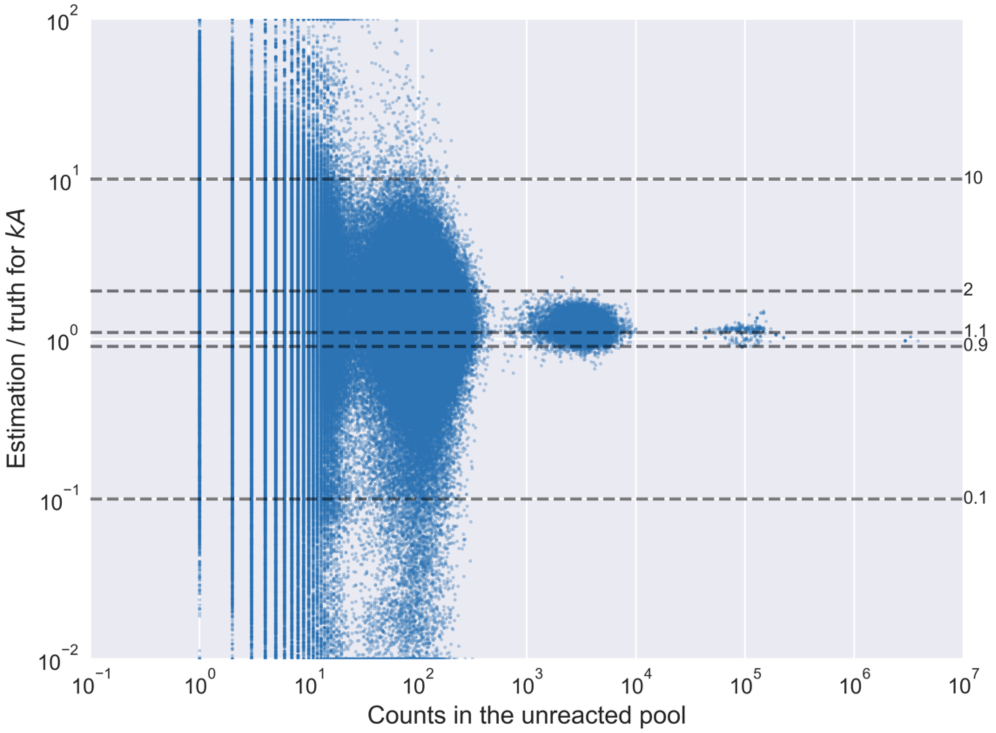

Figure S21: Accuracy and precision of kA estimation varies with kA and number of counts in the unreacted sample. A heatmap was constructed using simulated data with known true kA, for (A) mean fold error (accuracy) and (B) mean fold range (precision) for kA estimation, binned over various true kA and counts in the unreacted sample. Fold error is the ratio between the point estimate and the true value or the reciprocal of this ratio, whichever is greater than 1. Fold range is the ratio between the 97.5-percentile estimate and 2.5-percentile estimate, indicating the range of uncertainty. It can be seen that low activity sequences can be reasonably well-estimated at higher counts. Specifically, 7089 simulated sequences had $kA < 1$ $\text{min}^{-1}\text{M}^{-1}$ and counts > 1000. These sequences showed a mean fold error of 1.26 ± 0.17 (s.d.) between the point estimate and the true kA (accuracy) and a 2.04 ± 0.53 (s.d.) fold range for kA estimation (precision). Hexagonal bins containing at least 10 sequences are shown.

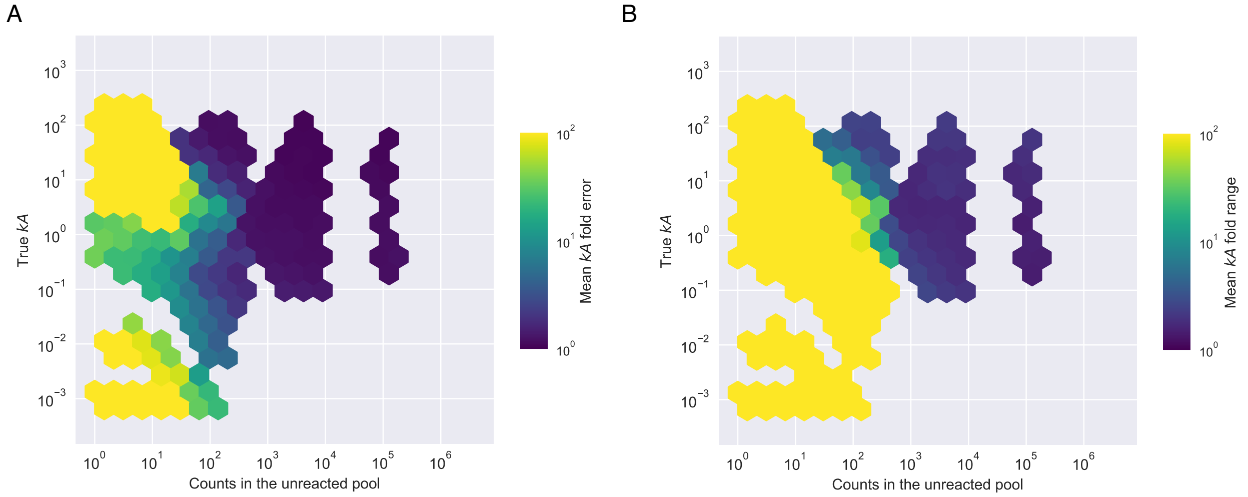

Figure S22. Pool evenness for the pool enriched by selection and the designed variant pool. Pool evenness is evaluated by normalized entropy ($-\sum_{i=1}^{N} p(i) \log p(i) / \log N$, where $p(i)$ is the fraction of sequence i and N is the number of unique sequences) for sequences with various minimum count thresholds in the unreacted samples. The variant pool is more even (higher normalized entropy) than the enriched pool.

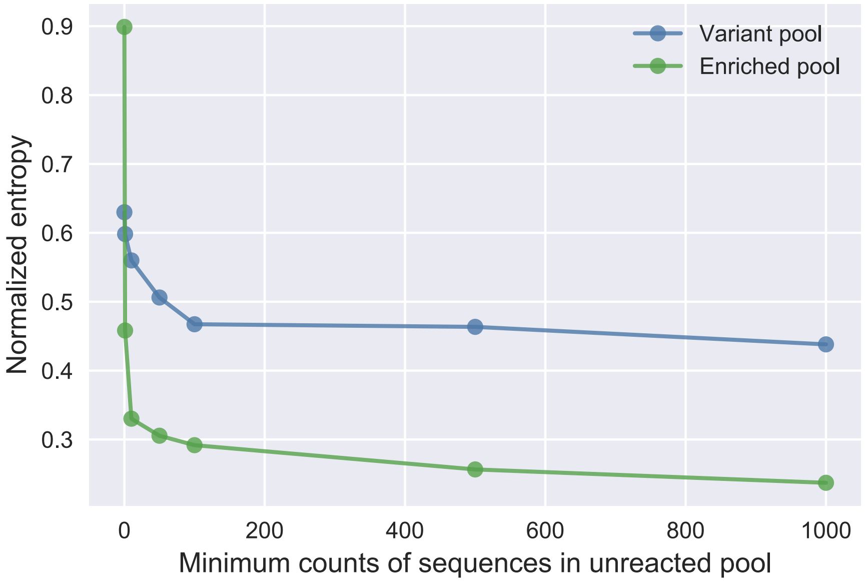

1. Pressman,A.D., Liu,Z., Janzen,E., Blanco,C., Müller,U.F., Joyce,G.F., Pascal,R. and Chen,I.A. (2019) Mapping a Systematic Ribozyme Fitness Landscape Reveals a Frustrated Evolutionary Network for Self-Aminoacylating RNA. *J. Am. Chem. Soc.*, **141**, 6213–6223.
